# Preformulation Study of Carbamazepine Orally Disintegrating Tablets for Pediatric Patients Using Direct Compression and the SeDeM Diagram Tool: A Quality by Design Approach

**DOI:** 10.3390/pharmaceutics17050624

**Published:** 2025-05-08

**Authors:** Ricard Canadell-Heredia, Khadija Rouaz-El-Hajoui, Natalia Franco-Piedrahita, Pilar Pérez-Lozano, Marc Suñé-Pou, Josep María Suñé-Negre, Encarna García-Montoya

**Affiliations:** 1 Department of Pharmacy and Pharmaceutical Technology and Physical Chemistry, Faculty of Pharmacy and Food Sciences, University of Barcelona, Av. Joan XXIII, 27-31, 08028 Barcelona, Spain; rcanadellh@gmail.com (R.C.-H.); nataliai.francop@ub.edu (N.F.-P.); perezlo@ub.edu (P.P.-L.); marcsune@ub.edu (M.S.-P.); jmsune@ub.edu (J.M.S.-N.); 2 Pharmacotherapy, Pharmacogenetics and Pharmaceutical Technology Research Group, Bellvitge Biomedical Research Institute (IDIBELL), Av. Gran via de l’Hospitalet, 199-203, 08090 Barcelona, Spain

**Keywords:** pediatric population, tablets, benign epilepsy, quality by design, SeDeM diagram

## Abstract

**Background/Objectives:** Carbamazepine is widely used as a first-line treatment for pediatric patients with benign epilepsy. However, most commercial formulations have doses of 100 mg or higher, limiting their suitability for pediatric use. The aim of this study was to develop mini orally disintegrating tablets (ODTs) containing 50 mg of carbamazepine, utilizing direct compression technology, specifically tailored to meet the unique needs of pediatric patients. **Methods:** The development was carried out following a Quality by Design (QbD) approach, beginning with preformulation studies using the SeDeM expert system. Various co-processed excipients (PROSOLV^®^ ODT and PARTECK^®^ ODT) and non-co-processed excipients (L-HPC LH11 and L-HPC NBD-022) were evaluated. Additionally, modifications to the radius parameter of the SeDeM expert system were investigated to improve formulation design. **Results:** Optimized Formulations 13 and 14 achieved disintegration times below 1 min, hardness values between 25 and 60 N, and friability under 1%, fulfilling the predefined Critical Quality Attributes (CQAs). Tablets were successfully produced with a diameter of 5 mm and a weight below 100 mg. Moreover, reducing the SeDeM incidence radius from 5.0 to values between 4.0 and 3.5 proved viable, enabling the inclusion of excipients previously considered unsuitable and broadening formulation options without compromising quality. **Conclusions:** This study demonstrates the feasibility of producing small, fast-disintegrating, and mechanically robust 50 mg carbamazepine ODTs tailored for pediatric patients. It also validates the adjustment of SeDeM parameters as an effective strategy to expand excipient selection and enhance formulation flexibility in pediatric drug development.

## 1. Introduction

Carbamazepine (C_15_H_12_N_2_O) is a tricyclic compound effective against partial seizures, with or without secondary generalization. First discovered in 1953, it was marketed in 1962 for treating trigeminal neuralgia and has been used as an anticonvulsant and antiepileptic in the United Kingdom since 1965, while it was approved in the United States since 1974 [1]. Carbamazepine is considered a first-line therapy in pediatric benign epilepsy [2,3].

Currently, the European Medicines Agency (EMA) and the Food and Drug Administration (FDA) are working on optimizing pediatric formulations, recognizing that children require formulations adapted to their specific needs. Although carbamazepine is not currently available in orally disintegrating tablets (ODTs), its use in pediatric and geriatric populations could improve administration and tolerance [4,5]. Dosing in children, according to the World Health Organization (WHO), begins at 5 mg/kg/day and is progressively adjusted up to a maximum of 20 mg/kg/day [6]. It is commercially available as oral suspension, tablets, and chewable tablets [2], though these formulations are primarily designed for adults, requiring dosage adjustments based on the child’s weight and age, which could pose potential risks to safety and stability [7]. The present study aims to develop 50 mg ODTs, enabling flexible dose adjustments. For example, for a child with an average weight of 20 kg, the minimum dose would be one tablet, and the maximum dose would be four tablets, to be administered according to the prescribed regimen. It is worth mentioning that the decision to develop 50 mg tablets is based on the FDA’s initial dosing recommendation for patients aged 6 to 12 years, which is 50 mg in four divided doses or 100 mg twice daily for the treatment of epilepsy with carbamazepine [8,9].

The development of ODTs for children faces challenges such as the low compressibility of carbamazepine. This study examines its formulation via direct compression, evaluating its galenic properties using the SeDeM expert system to optimize the design of 50 mg pediatric tablets, thereby facilitating therapeutic adherence and age-appropriate dosing [10,11].

To analyze carbamazepine’s compressibility properties for direct compression, the SeDeM expert system is used [12]. The system is a tool that characterizes powdered substances based on 12 parameters grouped in incidences as dimensions, compressibility, flowability, lubricity/stability, and lubricity/dose [12,13,14,15]. The information gathered from the SeDeM system helps identify favorable properties and weaknesses that need addressing if tablets by direct compression are to be developed. This tool facilitates the successful design of tablets by avoiding unnecessary studies and reducing development lead times, providing accurate knowledge in the initial stages of development. Additionally, the SeDeM expert system offers a mathematical equation for defining tablet formulation to be manufactured by direct compression in a straightforward manner, minimizing the required amount of excipient in the formulation [16,17]. Such formulations reduce costs in development, particularly for pediatric products where some active pharmaceutical ingredients may not be economically viable for industrial development and manufacturing.

Following the SeDeM system procedure, various excipients commonly used for producing tablets by direct compression have been characterized. These include low-substituted hydroxypropyl cellulose (L-HPC LH11 and L-HPC NBD-022), which is primarily used as a binder but may also act as a disintegrant by inducing swelling of the tablet. Additionally, co-processed excipients have been considered, such as a mixture of microcrystalline cellulose, colloidal silicon dioxide, mannitol, fructose, and crospovidone (PROSOLV^®^ ODT) and a mixture of D-mannitol and sodium croscarmellose (PARTECK^®^ ODT). These co-processed excipients serve various functions, including diluent, binder, and disintegrant. This selection differs from the excipients reported in previous studies, which mainly focused on individual disintegrants, such as crospovidone [18], a mixture of mannitol, crospovidone, and polyvinyl acetate (Ludiflash^®^), crospovidone and croscarmellose (Ac-Di-Sol^®^) [19,20,21], and sodium starch glycolate (Explotab^®^) [21].

The first objective of this study was to characterize carbamazepine and the chosen excipients to establish different formulations using the SeDeM methodology. The second objective was to define the compressibility behavior of the blends (API + excipients) proposed under the SeDeM system framework [12,14,16,22,23,24,25,26,27,28], aiming to determine a new value for the average incidence radius (R) that differs from the currently indicated value. Finally, the last objective was to conduct SeDeM characterization of the most viable formulas and determine their suitability for direct compression to obtain pediatric tablets for patients aged 6 years or older, of low weight and a diameter not exceeding 7 mm [29,30].

## 2. Materials and Methods

### 2.1. Material

Carbamazepine (CAS No. 298-46-4) was purchased from CTX Lifesciences Pvt. Limited, Surat, Gujarat. L-HPC LH11 (low-substituted hydroxypropylcellulose, CAS No. 9004-64-2) and L-HPC NBD022 (low-substituted hydroxypropylcellulose) were purchased from SHINETSU, Tokyo, Japan. PARTECK^®^ ODT (a mixture of D-mannitol (CAS No. 69-65-8) and sodium croscarmellose (CAS No. 74811-65-7)) was purchased from MERCK, Darmstadt, Germany. PROSOLV^®^ ODT (a mixture of microcrystalline cellulose (CAS No. 9004-34-6), colloidal silicon dioxide (CAS No. 7631-86-9), mannitol, fructose, and crospovidone (CAS No. 9003-39-8)) was purchased from JRS PHARMA, Rosenberg, Germany. Talc (CAS No. 14807-96-6), magnesium stearate (CAS No. 557-04-0), and colloidal silicon dioxide (Aerosil^®^) were purchased from Fagron Ibérica, Barcelona, Spain.

To formulate the API, excipients were chosen based on their direct compression characteristics as well as their properties as disintegrating and diluting agents.

### 2.2. Methods

#### 2.2.1. QbD Application to the Formulation Development

Following the guidelines outlined in the ICH Q8 (R2) standard [31], a fundamental Quality Target Product Profile (QTPP) and Critical Quality Attributes (CQAs) have been established. Subsequently, a risk analysis, according to ICH Q9 [32], will be carried out to identify critical parameters within the blending and compression process.

#### 2.2.2. Crystal X-Ray Diffraction Method

XRD analysis was conducted utilizing an X’Pert Pro MPD X-ray diffractometer (PAN-alytical, Malvern, UK). The analyzed sample consisted of pure carbamazepine powder (from III), which was encapsulated between polyester films with thicknesses of 3.6 micrometers. The equipment was configured with a convergent beam, an elliptic mirror, and a transmission geometry with flat samples sandwiched between low-absorbing films. Cu Kα radiation (λ = 1.5418 Å) was utilized, with operating parameters set at 45 kV and 40 mA. The PIXcel detector was configured with an active length of 3.347° 2ϴ, scanning a range from 2 to 60° 2ϴ with a step size of 0.026° 2ϴ and a measuring time of 300 s per step.

#### 2.2.3. Determination of Particle Size Distribution (PSD)

The particle size distribution was determined according to the general method “2.9.31. Particle size analysis by laser light diffraction” of the Eur. Ph. [33], employing a MASTERSIZER 2000 instrument (Malvern, UK) equipped with a module for the wet process determination (HYDRO). The test conditions involved the use of water as the dispersant, with a refractive index of 1.33, and a stirrer speed set at 2500 rpm. Three measurement cycles were conducted, each lasting 6 s and resulting in 6000 measurement snapshots. Additionally, background measurements were performed over 12 s, resulting in 12,000 background snapshots.

#### 2.2.4. Powder Characterization Using SeDeM System

The SeDeM method has been applied to evaluate the appropriateness of both the active pharmaceutical ingredient and excipient for direct compression, as well as to determine the suitability of powder mixtures for direct compression purposes. To assess the powder, 12 different parameters have been delineated and categorized into 5 incidence factors, based on the physical characteristics of the powder and the functionality of the drug. The numeric value of each incidence factor represents the mean value derived from the associated parameter values, known as the average incidence radius. Subsequently, the factors included in each incidence will be outlined.

*Dimensional Incidence Factor*

The dimensional incidence factor influences the size of the tablet and its ability to stack effectively. Additionally, these tests contribute to the calculation of other mathematical indexes for determining compressibility parameters. The associated parameters are as follows:

- **Bulk density (*Da*)**: Bulk density was determined according to monograph 2.9.34 of the Eur. Ph. [34]. Approximately 100 g of sample (or an adjusted amount to obtain a volume between 50 and 250 mL) was carefully poured into a graduated cylinder without compacting the powder. The initial volume (*V_o_*) occupied by the sample was recorded without applying any mechanical treatment. Bulk density was then calculated using the following equation:
  (1) $$Da=\frac{m}{V_{o}}$$
  where *m* is the mass (g) of the sample and *V_o_* the initial apparent volume (mL).

- **Tapped density (*Dc*)**: Tapped density was measured using the same setup as for bulk density, following the procedure described in monograph 2.9.34 of the Eur. Ph. [34]. After recording the initial volume, the cylinder was tapped using an automatic volumeter (SBS Volumenometer) with 10, 500, and 1250 taps. The apparent volume was recorded after each tapping cycle. If the difference between the volumes measured after 500 and 1250 taps exceeded 2 mL, an additional 1250 taps were applied, bringing the total to 2500. The final tapped density (*V*_1250_ or *V*_2500_) was used to calculate the tapped density using the following equation:
  (2) $$Dc=\frac{m}{V_{1250}}\;or\;Dc=\frac{m}{V_{2500}}$$
  where *m* is the mass (g) of the sample and *V*_1250_ and *V*_2500_ are the compacted apparent volumes (mL) after 1250 and 2500 strokes, respectively.

*Compressibility Incidence Factor*

The compressibility incidence factor affects the compressibility of the powder. The associated parameters are as follows:

- **Interparticle porosity (*Ie*)**: The interparticle porosity of the powder mixture is calculated from the following Equation (3).
  (3) $$\mathrm{Ie}=Dc-\frac{Da}{Dc}\times Da$$

- **Carr index (*IC*)**: This parameter is calculated using Equation (4).
  (4) $$IC=\left(Dc-\frac{Da}{Dc}\right)\times 100$$
  In both equations, *Dc* represents the tapped density (g/mL) and *Da* represents the bulk density (g/mL) of the powder mixture.

- **Cohesion index (*Icd*)**: This index is determined by compressing the powder, preferably using an eccentric press. Initially, the mean hardness (N) of the tablets is calculated. If the raw powder cannot be compressed, 3.5% of the standardized lubricant mixture shown in Table 1 is added.

*Flowability/Powder Flow Incidence Factor*

This incidence factor impacts the flowability of the powdered substance during compression. The associated parameters include the following:

- **Hausner ratio (*IH*)**: Determines the easiness of flow of the studied sample. It is calculated from Equation (5), as outlined in Section 2.9.34 of the Eur. Ph. [34].
  (5) $$IH=\frac{Dc}{Da}$$
  where *Dc* represents the tapped density (g/mL) and *Da* represents the bulk density (g/mL) of the powder mixture.

- **Angle of repose (*α*)**: The angle is determined according to the method described in Section 2.9.36 of the Eur. Ph. [35]. It represents the angle of the cone formed when the product is passed through a funnel with specific dimensions: a funnel height of 9.5 cm, an upper diameter of spout of 7.2 cm, and an inner diameter at the bottom narrow end of the spout of 1.8 cm. The funnel (ANORSA reference X5992) is positioned on a stand 20 cm above the tablet surface, centered on a millimeter grid sheet where two intersecting lines mark the center. The narrow end of the funnel nozzle is capped and leveled with the sample by running a spatula along the funnel sides. Upon removing the stopper, the powder falls onto the millimeter sheet. The four radii at the base of the cone are measured using a sliding caliper, and their mean value (*r*) is calculated. Additionally, the height (*h*) of the cone is measured. Finally, the tangent of the cone angle (*α*) is determined using Equation (6).
  (6) $$Tan\;\left(\alpha \right)=\frac{h}{r}$$
  where *h* is cone height and *r* is the average value of the four radii.

- **Powder flow (*t″*)**: This parameter, expressed in seconds and tenths of a second per 100 g of sample, was determined according to the method described in Section 2.9.16 of the Eur. Ph. [36]. The flowability was assessed by measuring the time required for a powder to flow through a standardized funnel. The equipment used included an ANORSA funnel (reference X7705), a metal stand with a clamp, and a stopwatch. The funnel’s orifice was initially sealed with paper, and 100 g of sample was poured into the funnel. Once filled, the paper plug was removed, and the time taken for the entire sample to flow through the funnel was recorded using the stopwatch. The procedure was repeated three times, and the average of the three measurements was reported as the result.

*Lubricity/Stability Incidence Factor*

This incidence factor affects the lubricity and long-term stability of tablets. The associated parameters include the following:

- **Loss on drying (*%HR*)**: This parameter is determined according to the method outlined in Section 2.2.32 in the Eur. Ph. [37]. The sample is dried in an oven at 105 °C ± 2 °C until a constant weight is obtained.
- **Hygroscopicity (*%H*)**: This parameter quantifies the percentage increase in sample weight after exposure to a humidifier set at a relative humidity of 76% (±2%) and a temperature of 22 °C ± 2 °C for 24 h.

*Lubricity/Dosage Incidence Factor*

This incidence factor influences the lubricity and dosage of tablets. The associated parameters include the following:

- **Particle size < 50 mcm (*%Pf*)**: The percentage of fine particles (<50 µm) is determined using a sieve test following the general method 2.9.12 of the Eur. Ph. [38]. The reported value represents the percentage of particles that pass through a 0.05 mm sieve when vibrated for 10 min at speed 10 (CISA^®^ vibrator).
- **Homogeneity index (*Iθ*)**: This index is calculated according to the general method 2.9.12 of the Eur. Ph. [38] for particle size determination by means of the sieve test. The grain size of a 100 g sample is measured by subjecting a sieve stack to vibration for 10 min at the speed of 10 (CISA vibrator). The percentage of product retained in each sieve is calculated, and the amount that passes through the 0.05 mm sieve is measured. The sieve sizes used are 0.355 mm, 0.212 mm, 0.100 mm, and 0.05 mm. Equation (7), described in previous works [10,11], is applied.
  (7) $$I\theta =\frac{F_{m}}{100+\left(d_{m}-d_{m-1}\right)F_{m-1}+\left(d_{m+1}-d_{m}\right)F_{m+1}+\left(d_{m}-d_{m-2}\right)F_{m-2}+\left(d_{m+2}-d_{m}\right)F_{m+2}\ldots +\left(d_{m}-d_{m-n}\right)F_{m-n}+(d_{m+n}-d_{m})F_{m+n}}$$
  where:
  *Iθ*: Relative homogeneity index. Particle size homogeneity in the range of the fractions studied.
  *Fm*: Percentage of particles in the majority range.
  *Fm* − 1: Percentage of particles in the range immediately below the majority range.
  *Fm* + 1: Percentage of particles in the range immediately above the majority range.
  *n*: Order number of the fraction studied under a series, with respect to the major fraction.
  *dm*: Mean diameter of the particles in the major fraction.
  *dm* − 1: Mean diameter of the particles in the fraction of the range immediately below the majority range.
  *dm* + 1: Mean diameter of the particles in the fraction of the range immediately above the majority range.

Once the parameter values were obtained using the methodology described above (SeDeM Diagram), they were transformed into radius values (*r*) for graphical representation. This conversion allows for data standardization and facilitates comparison regardless of the original measurement units.

The transformation equations used follow those proposed by Suñé-Negre et al. [15,17], as well as additional guidelines from other relevant studies [14,22,28,39]. These equations, summarized in Table 2, involve scaling each parameter to a dimensionless value between 0 and 10. To apply these equations, numerical limits were first established for each of the 12 evaluated parameters. These limits were based either on values recommended in the Handbook of Pharmaceutical Excipients [40] or, where necessary, on experimental data generated in this study. Values falling below 0 were adjusted to 0, and those above 10 were limited to 10, in accordance with SeDeM Diagram conventions. This process ensured that the final diagram accurately reflected the relative suitability of each parameter for direct compression and provided a clear and quantitative overview of the formulation’s compressibility profile.

To numerically assess the product’s appropriateness for direct compression, the following indexes are calculated:

- **Parametric profile index (*IPP*)**. This index represents the mean value of all calculated parameters, with an acceptability limit set at *r* ≥ 5.
- **Good compression index (*IGC*)**. This index is calculated from Equation (8), where f is the reliability factor, determined by the ratio of polygon area to circle area. A *GCI* value greater than 5 is necessary to permit direct compression processing.
  (8) GCI=IPP×f
  where *IPP* is the parametric profile index and *f* is the reliability limit for *IPP* would be equal to or higher than 5. *f* can be calculated by using Equation (9).
  (9) $$f=\frac{polygon\;area}{circle\;area}$$

It is necessary to note that the SeDeM Diagrams featured in this paper were generated using a validated Microsoft Excel^®^ spreadsheet developed by the Service of Development of Medicines (SDM) at the Faculty of Pharmacy and Food Sciences, University of Barcelona.

#### 2.2.5. Formulation Design Under the SeDeM Perspective

To perform the compressibility study of carbamazepine with the various disintegrants selected, several formulations were developed. For 12 parameters, *f* is equal to 0.952. The quantity of each excipient to be included was determined using the following mathematical equation [17]:

(10) $$CP=100-\left[\left(\frac{RE-R}{RE-RP}\right)\times 100\right]$$

where:

*CP*: Percentage of excipient to be added.

*RE*: Excipient average incidence radius value for compressibility.

*R*: Average incidence radius value to be obtained in the blend.

*RP*: API average incidence radius value for compressibility.

Normally, the values investigated for the target average incidence radius value in the blend (R) follow a decreasing series (5.0, 4.5, 4.0, and 3.5) for each excipient, although historically, 5.0 has been considered the optimal value [15,17,30]. In this study, to obtain the most desirable percentages, the target will be reduced in order to achieve smaller tablet dimensions.

#### 2.2.6. Blends Preparation

Various blends were prepared using the following method (see Table 3 for composition details): the raw materials were individually weighed into polyethylene bags. Subsequently, each component was passed through a 0.6 mm sieve to ensure uniform particle size distribution. The sieved powders were then transferred to an appropriate container and blended for 25 min at 20 rpm in the Glatt biconical mixer (Glatt^®^ Labortechnic, Barcelona, Spain). Finally, magnesium stearate was added to the blend and mixed for an additional 3 min at the same speed.

#### 2.2.7. Tablet Preparation

The various blends under investigation were compressed in an eccentric compression machine (Bonals^®^, Cornellà de Llobregat, Spain), employing different sets of flat-faced round punches with diameters ranging from 5 to 13 mm to produce tablets containing 50 mg of carbamazepine, with a weight that varied depending on the percentage of excipient added. It is worth mentioning that all compressions are performed using the direct compression process.

#### 2.2.8. Tablet Characterization

*Weight Variation*

Twenty tablets were compressed and weighed for each formulation. The average weight was calculated, and individual weights were compared with the average according to the general method described in Section 2.9.5 of the Eur. Ph. [41].

*Hardness*

To characterize the mechanical strength of the tablets, ten units from each formulation were tested using a calibrated durometer (Dr. Schleuniger^®^, Solothurn, Switzerland), according to the general method specified in Eur. Ph. 2.9.8. [42]. Each tablet was placed between two plates, and force was applied until the tablet fractured. The breaking force, expressed in Newtons (N), was recorded for each unit. The average value of the ten determinations was considered representative for each formulation.

*Friability*

Tablet friability, which assesses the ability of tablets to withstand abrasion during handling, was evaluated using a calibrated friabilometer (Dr. Schleuniger^®^, Solothurn, Switzerland), following the general procedure described in Eur. Ph. 2.9.7. [43]. A pre-weighed sample of tablets (equivalent to 6.5 g to 6.8 g, or approximately 10 tablets) was rotated at 25 rpm for 4 min, completing 100 revolutions. After testing, the tablets were dedusted and reweighed. Friability was calculated as a percentage loss in tablet weight using Equation (11).

(11) $$\mathrm{Friability}\;(\%)=\frac{W1-W2}{W1}\times 100$$

where *W1* and *W2* are the weights of the tablets before and after the test.

*Disintegration time*

The disintegration time was determined following the general method outlined in Eur. Ph. 2.9.1 [44], utilizing a calibrated disintegration apparatus (Schleuniger^®^ Pharmatron DTG3000, Solothurn, Switzerland). Deionized water at a volume of 700 mL and a temperature of 37 °C ± 2 °C served as the disintegration medium. Each tablet was positioned within its respective disintegration basket alongside a disk. Disintegration time was noted when all tablet fragments had completely passed through the mesh of the disintegration basket.

#### 2.2.9. Product Characterization: Initial Risk Assessment

Following the guidelines outlined in the ICH Q8 (R2) [31] standard, a fundamental Quality Target Product Profile (QTPP) has been established. The initial characteristics of the product under consideration in this study are presented in Table 4.

The dose specified in the QTPP for orally disintegrating tablets of carbamazepine is 50 mg per tablet. This dosage is deemed appropriate for pediatric administration, considering that initial doses of 100 mg, taken 1–2 times daily, are recommended [6,45]. By manufacturing tablets at this dosage, the aim is to potentially modify the current administration regimen, enhance tolerance, or introduce new combinations with other drugs commonly utilized in pediatric epilepsy treatment.

## 3. Results and Discussion

To ensure the quality characteristics of the tablets produced in this initial development phase, which are subsequently associated with the attributes of the final formulations [46,47,48,49,50,51], a comprehensive assessment of Critical Quality Attributes (CQAs) and risk analysis, as detailed in Table 5, has been established.

The risk analysis of the formulation was conducted by considering the uncontrollable critical material attributes, which depend on the quality of the raw material, such as polymorph form, moisture, and particle size for carbamazepine, as determined through the application of the SeDeM method [52,53,54,55,56,57]. Critical material attributes related to the disintegrant and lubricant were analyzed in a general manner, as their impact on the formulation is established by the SeDeM method. The chosen CQAs included flow (which affects weight and process characteristics), hardness (which impacts friability, disintegration, and dissolution in the final formula), friability (which influences packaging, transport, and patient handling), and disintegration time (which affects tablet dissolution).

The results of this risk analysis underscore the necessity to define the characteristics of the polymorph type and the particle size utilized during the preformulation studies of carbamazepine (API), due to their significant impact on the final formulation. Carbamazepine is an active pharmaceutical ingredient with well-documented polymorphism, comprising at least four anhydrous forms (I–IV) and one dihydrate, each with distinct thermodynamic stability and dissolution behavior. Form III, employed in this study, was selected based on its higher physical stability and lower tendency to undergo polymorphic transitions during manufacturing or storage. Although form II exhibits enhanced dissolution, its reduced stability increases the risk of transformation under mechanical stress, potentially affecting flow and compressibility [8,55,56,57,58]. These considerations underscore the importance of selecting a stable polymorph and maintaining its consistency throughout manufacturing to minimize variability in Critical Quality Attributes [54].

While the lubrication step is a factor to consider, it was evaluated according to the SeDeM method guidelines, which have demonstrated satisfactory rheological properties in prior studies [17], thereby indicating a low level of risk. However, it would be prudent to investigate the influence of the lubricant on flow properties and its impact on the uniformity of tablet mass in subsequent stages.

### 3.1. Carbamazepine Characterization

The X-ray diffraction analysis was conducted on the carbamazepine. As published by Adam I.G. et al. [58], polymorphic form III exhibits four distinctive points of 2ϴ in its X-ray spectrum, with values at 15.36, 19.56, 25.00, and 27.47. The obtained results confirm that carbamazepine examined in this study crystallized according to the monoclinic (p-monoclinic) system, specifically as polymorph III (see Figure S1 in the Supplementary Materials).

Additionally, the particle size distribution of the carbamazepine batch used was determined, indicating that 10% of the particles are smaller than 6.95 µm, 50% are smaller than 46.47 µm, 90% are smaller than 143.93 µm, and 100% are smaller than 447.74 µm, as illustrated in Figure S2 of the Supplementary Materials.

**Figure 1:**
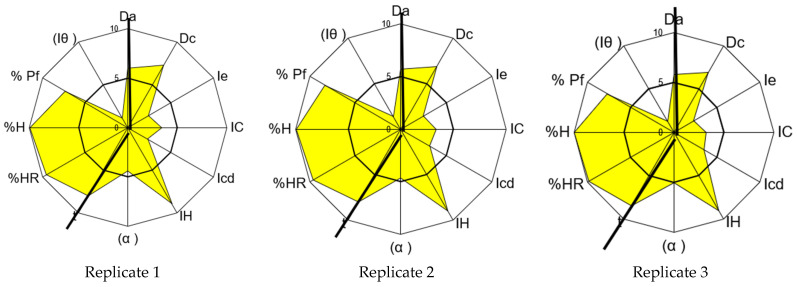
SeDeM graphical representations for carbamazepine (three replicates of the same batch).

Following the determination of the non-controllable critical material attributes for carbamazepine, its characterization was conducted using the SeDeM method. The obtained results (three replicates) are presented in Table 6 and Figure 1.

The analysis of the results indicates an average global compression index (GCI) for carbamazepine of 5.72, consistent with the value of 5.28 reported by Campiñez M.D. et al. [59]. According to the SeDeM system, this demonstrates the suitability of carbamazepine for direct compression tablet technology. However, a more detailed examination of this result reveals a deficiency in terms of the compressibility incidence factor of the API. Parameters representative of compressibility (porosity between particles, Carr’s index, and Cohesion index) show an average incidence value of 2.92, which is lower than 5.0, indicating potential difficulties in manufacturing via direct compression.

Although the analyzed carbamazepine exhibits a low percentage of particles smaller than 50 µm, according to the SeDeM Diagram, it is deemed acceptable (mean r = 7.80). However, the homogeneity index of the particle size is deficient (mean r = 1.25), indicating high dispersion in the distribution of particle sizes. This results in a moderately acceptable lubricity/dosage incidence factor of the SeDeM Diagram (mean r = 4.53), suggesting no significant impact on the correct direct compression process.

To address this deficiency in the compressibility index, it is proposed to incorporate a disintegrant with higher compressibility index values, thereby correcting this deficit [16]. Other incidence factors are considered acceptable and do not require correction, with values of 5 or higher.

### 3.2. Excipient Characterization

**Figure 2:**
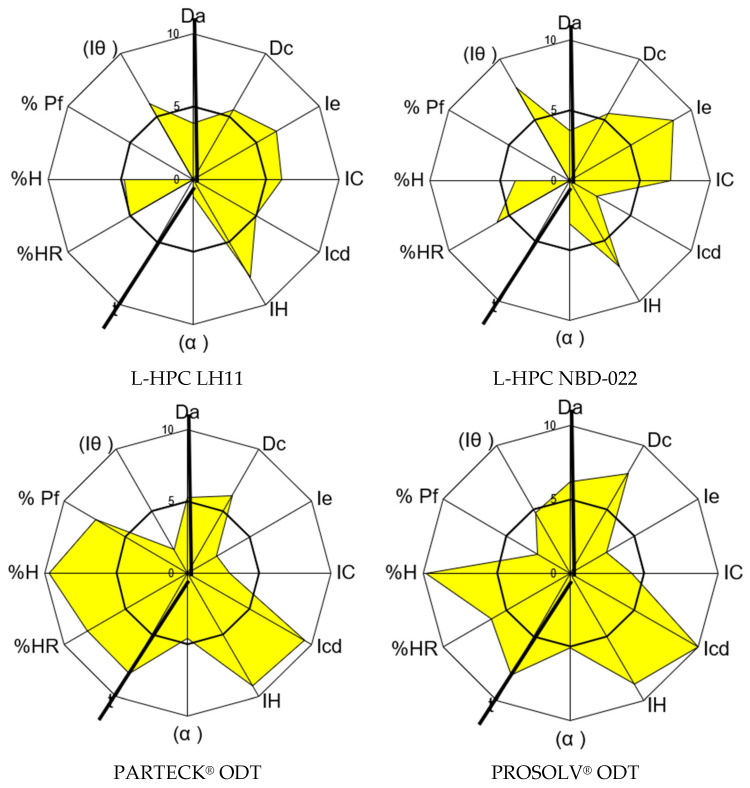
SeDeM graphical diagrams of the disintegrants.

The disintegrants chosen to address the observed compressibility index of carbamazepine include L-HPC LH11, L-HPC NBD022, PROSOLV^®^ ODT, and PARTECK^®^ ODT. All these options demonstrate favorable characteristics for direct compression and are well-established for their disintegration function. The results obtained for each disintegrant are presented in Table 6 and depicted in Figure 2.

L-HPC LH11 and L-HPC NBD022 are low-substituted hydroxypropyl celluloses recognized for their suitability in formulating orodispersable tablets due to their rapid disintegrating and compressibility properties. Both can be incorporated into formulations in quantities ranging from 5 to 50%. The d90 for L-HPC LH11 is 150–200 microns, while for L-HPC NBD022, it ranges from 70 to 130 microns [60].

Analysis of the SeDeM characterization results for both L-HPC LH11 and L-HPC NBD022 indicates an average GCI below 5 due to observed deficiencies in flowability/powder flow and lubricity/dosage incidence factors. Despite their inability to be compressed alone by direct compression, their compressibility incidence factor demonstrates values of 5.90 for L-HPC LH11 and 6.00 for L-HPC NBD022, which are sufficient to correct the observed deficiency in the compressibility factor of carbamazepine.

PARTECK^®^ ODT as well as PROSOLV^®^ ODT are commercially processed products designed to enhance the compressibility of mixtures. Both were selected for their favorable disintegrating and compression properties. SeDeM characterization results show a GCI value higher than 5 for both mixtures, indicating suitability for direct compression under the SeDeM method. However, their compressibility incidence factor is lower compared to non-co-processed excipients, with values of 4.91 for PARTECK^®^ ODT and 5.66 for PROSOLV^®^ ODT, due to suboptimal Carr index and interparticle porosity values. Nevertheless, both mixtures exhibit promising cohesion capacity and favorable flow properties, making them attractive candidates, especially if the final mixture will be compressed without prior granulation.

In summary, all four excipients demonstrate compressibility incidence factor values higher than that of carbamazepine (i.e., 2.92). Therefore, they are deemed suitable excipients for correcting this API deficiency parameter.

### 3.3. Compressibility Approach

Taking into consideration the methodology outlined by the SeDeM method [17,18,19,20,21,22,23,24,25,26,27,28] and a theoretical value of 5.0 for the radius of compressibility incidence in a blend of carbamazepine plus excipients (R), the resulting blend would be deemed suitable for direct compression.

Upon analyzing the results of parameters corresponding to the interparticle porosity, the Carr index, and the Cohesion index of the API and the four selected excipients, it can be inferred that the blends formed with them would permit direct compression, in accordance with the mathematical equation (Equation (1)).

To optimize the proportion of excipient to be added to carbamazepine and to determine the minimum required excipient percentage, various minimum values of compressibility incidence radius were examined. It is worth noting that the focus is on the minimum number of excipients, as this would allow the production of the smallest possible tablets, making them suitable for a wider range of pediatric patient ages. Previous studies have demonstrated that slightly lower rates than 5.0 also yielded compressible blends [12,13,14,17,23,61].

Consistent with ICH Q8 guidelines [31], in establishing the design and control parameters, the minimum value that could be applied was confirmed, considering that carbamazepine exhibits a GCI below 5. The newly selected minimum values were 4.5, 4.0, and 3.5. Consequently, the mathematical equation (Equation (1)) was applied to ascertain the percentage of each excipient and carbamazepine for each chosen GCI.

The formula composition for all the R values was designed, maintaining a fixed dose of 50 mg of carbamazepine while adjusting the final tablet weight. The active pharmaceutical ingredient and the other excipients (talc, colloidal silicon dioxide (Aerosil^®^), and magnesium stearate) were incorporated as specified in the SeDeM method [10,12,15] to facilitate a standard direct compression process. The percentages of excipients and the formulation composition are presented in Table 3.

These findings are in line with recent developments in the formulation of pediatric ODTs of carbamazepine. Canadell-Heredia et al. [2] successfully applied the SeDeM method to formulate 50 mg ODTs using L-HPC LH11 and NBD022, obtaining tablets with appropriate hardness and disintegration profiles without the need for prior granulation. Their study also confirmed the suitability of polymorph III and highlighted the need to correct the compressibility deficiency of carbamazepine to enable direct compression. The present work supports these conclusions and further demonstrates that reducing the incidence radius to values as low as 3.5 remains feasible for producing mini ODTs suitable for pediatric use.

Moreover, from a clinical perspective, the relevance of adapting carbamazepine formulations to pediatric needs is supported by the findings of Jung et al., who demonstrated that carbamazepine, when administered as monotherapy in children with focal epilepsy, was both safe and effective, showing no negative impact on neuropsychological function [62]. Thus, the development of child-adapted solid dosage forms such as the mini ODTs described in this study could significantly improve treatment adherence and ease of administration in the pediatric population.The average weight and hardness values obtained after compressing the different theoretical blends are shown in Table 7. The tablets produced exhibit correct compression and confirm a proper shape without adhesion, indicating their suitability.

Analysis of the hardness values as a quality parameter, with the highest value expressed for each formula studied, reveals that the results obtained with the maximum disintegrant compensation (R value equal to 5) yield the best hardness values (see Table 7). This finding aligns with the SeDeM method. Tablet hardness values for other selected compressibility incidence factors show a decreasing trend, as expected due to the reduction in the amount of corrective excipient.

**Figure 3:**
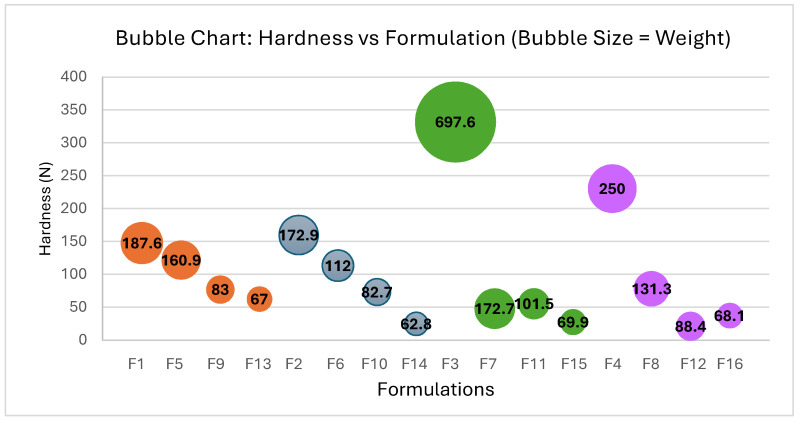
Correlation between hardness and Formulations F1–F16: impact of weight. Each color group represents formulations containing the same excipients in their composition: PARTECK^®^ ODT (F3, F7, F11, and F15), PROSOLV^®^ ODT (F4, F8, F12, and F16), L-HPC LH11 (F1, F5, F9, and F13) and L-HPC NBD022 (F2, F6, F10, and F14) (see Table 7).

Furthermore, the decline in hardness values observed in tablets formulated with the co-processed excipients, such as PARTECK^®^ ODT (F3, F7, F11, and F15) and PROSOLV^®^ ODT (F4, F8, F12, and F16), tends to be more pronounced compared to tablets formulated with the non-co-processed excipients, such as L-HPC LH11 (F1, F5, F9, and F13) and L-HPC NBD022 (F2, F6, F10, and F14) (see Figure 3).

The results indicate that the gradual reduction in R values to 4.5, 4.0, and 3.5 confirmed that, while none of the formulations encountered issues during the compression process, hardness decreases proportionally with the decrease in R value while still allowing for the proper formation of orally disintegrating tablets.

Considering the QTP approach, only tablets with an R value of 4.0 and 3.5 enable the production of tablets with a diameter smaller than 7 mm. However, formulas with an R value of 3.5 were chosen, as they allow the production of tablets with a diameter of 5 mm, which is predictably more acceptable among the pediatric population.

Additionally, the characterization of mixtures and tablets was conducted on formulas F13 to F16 to verify the remaining CQA, including friability and disintegration time. The results, as shown in Table 8, confirm the suitability of both the tablets and the mixture under study to produce orally disintegrating tablets.

The hardness values observed with an R value of 3.5 (see Table 8) are notably good for ODT formulation, as lower hardness contributes to faster disintegration, as demonstrated in Formulations F13 to F16. Among the formulas obtained with non-co-processed excipients, Formulation F14 exhibits the lowest friability, indicating better compaction that minimizes product loss during mechanical erosion. As expected, formula F13 shows higher friability due to the long, fibrous particles of the LH11 excipient, which hinder compaction of the mixture. Formulations obtained with co-processed excipients (F15 and F16) display acceptable hardness values, but their friability values suggest inadequate compaction in both cases.

Furthermore, the disintegration times for the four formulations reveal that Formulations F13 and F14 demonstrate the most favorable values, each disintegrating in under 1 min. Hence, it can be concluded that the most promising results for producing DC-ODT of carbamazepine are achieved with the excipient L-HPC NBD022 (F14). Following closely for faster disintegration is Formulation F13, using L-HPC LH11. However, to determine the definitive formulation for F13 and F14, further studies will be necessary to optimize the remaining incidence factors, particularly blend flowability, as indicated by the obtained Hausner’s ratio.

The distinct behavior of the disintegrants used enables differentiation between co-processed and non-co-processed disintegrants when mixed with carbamazepine. Co-processed agents exhibit superior compression results at R 5.0, whereas non-co-processed agents demonstrate better compression homogeneity.

**Figure 4:**
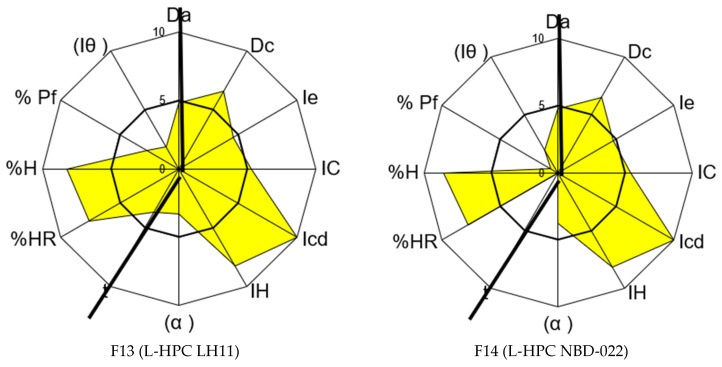
SeDeM graphical representations of Formulations F13 and F14.

The complete SeDeM characterization results for the Formulations F13 and F14, formulated with the non-co-processed excipient, are presented in Table 9 and Figure 4. In both formulations, the compressibility incidence value has increased to 6.63 and 6.73, respectively, surpassing the original value of 2.92 for carbamazepine. However, the good compression index (GCI) is lower than the original value obtained for the API, with values of 5.31 and 4.94 compared to the initial mean value of 5.72, which is high due to the incidence of lubricity/stability and flowability (see Table 9). This discrepancy is attributed to a decrease in the flow properties of the blends, highlighting the need to improve this characteristic in the final formulation. Nevertheless, this difference does not seem significant, as the values are close to 5 and do not affect compression.

## 4. Conclusions

In this study, two formulations for producing orally disintegrating tablets of carbamazepine using direct compression technology were proposed. A correlation between carbamazepine and the selected excipients was established, resulting in improved compressibility of the API. The results confirm the successful development of viable pediatric ODTs with a 5 mm diameter and low weight for a 50 mg dose, in line with the defined Critical Quality Attributes and risk analysis.

This study also evaluated the possibility of reducing the incidence radius (typically set at 5) to achieve smaller tablets more suitable for pediatric use. Mean incidence radius values between 4.0 and 3.5 were found to be appropriate, and the good performance of Formulations F13 and F14 supports this adjustment. These findings suggest that the current SeDeM expert system (R = 5.0) could be lowered, providing greater flexibility in future formulation development.

Finally, a reproducible formulation has been proposed that meets pediatric requirements. Given the lack of commercially available carbamazepine doses adapted to children and the scientific support for ODTs as a preferred option, this work contributes to addressing an important therapeutic gap.

## Figures and Tables

**Table 1:** Standardized lubricant mixture for Cohesion index study.

Components	Percentage (%)
Talc	2.36
Aerosil^®^ 200	0.14
Magnesium stearate	1.00

**Table 2:** Transformation equations for twelve parameters in the SeDem expert system.

Parameter	Symbol	Unit	Equation	Acceptable Ranges	Equation to Convert Values to SeDeM Radius Values
Bulk density	*Da*	g/mL	*Da* = m/V_0_	0–1	10 v
Tapped density	*Dc*	g/mL	*Dc* = m/V_1250_ *Dc* = m/V_2500_	0–1	10 v
Interparticle porosity	*Ie*	-	*Ie* = (*Dc* – *Dc*)/(*Dc* × *Da*)	0–1.2	10 v/1.2
Carr index	*IC*	%	*IC* = ((*Dc* − *Da*)/*Dc*) × 100	0–50	v/5
Cohesion index	*Icd*	N	Experimental	0–200	v/20
Hausner index	*IH*	-	*IH* = *Dc*/*Da*	3–1	(30–10 v)/2
Angle of repose	*α*	°	Experimental	50–0	10–(v/5)
Powder flow	*t″*	s	Experimental	20–0	10–(v/2)
Loss on drying	*%HR*	%	Experimental	10–0	10-v
Hygroscopicity	*%H*	%	Experimental	20–0	10–(v/2)
Particles < 50 µm	*%Pf*	µ	Experimental	50–0	10–(v/5)
Homogeneity index	*Iϴ*	-	*Iϴ* = *Fm*/(100 + Δ*Fmn*)	0–0.02	500 v

**Table 3:** Percentage of excipient suitable for direct compression of carbamazepine (API) according to the SeDeM model equation and formulas composition.

Mixture	Components	Percentage for Compressibility Radius 5.0				Percentage for Compressibility Radius 4.5				Percentage for Compressibility Radius 4.0				Percentage for Compressibility Radius 3.5			
		F1	F2	F3	F4	F5	F6	F7	F8	F9	F10	F11	F12	F13	F14	F15	F16
Mixture 1	Carbamazepine	26.70	28.97	7.23	20.59	43.48	45.20	28.69	38.84	60.26	61.44	50.15	57.08	77.04	77.67	71.61	75.33
	L-HPC LH11	69.80	-	-	-	53.02	-	-	-	36.24	-	-	-	19.46	-	-	-
	L-HPC NBD022	-	67.53	-	-	-	51.30	-	-	-	35.06	-	-	-	18.83	-	-
	PARTECK^®^ ODT	-	-	89.27	-	-	-	67.81	-	-	-	46.35	-	-	-	24.89	-
	PROSOLV^®^ ODT	**-**	-	-	75.91	-	-	-	57.66	-	-	-	39.42	-	-	-	21.17
	Talc	2.36	2.36	2.36	2.36	2.36	2.36	2.36	2.36	2.36	2.36	2.36	2.36	2.36	2.36	2.36	2.36
	Aerosil^®^	0.14	0.14	0.14	0.14	0.14	0.14	0.14	0.14	0.14	0.14	0.14	0.14	0.14	0.14	0.14	0.14
Mixture 2	Magnesium stearate	1.00	1.00	1.00	1.00	1.00	1.00	1.00	1.00	1.00	1.00	1.00	1.00	1.00	1.00	1.00	1.00

**Table 4:** Quality Target Product Profile (QTPP) for carbamazepine pediatric tablets.

QTPP Elements	Target
Dosage form	Orally disintegrating tablets
Route of administration	Oral
Dosage strength	50 mg
Shape	Tablet diameter < 7 mm
Disintegration time	Not more than 3 min
Pediatric population	Aged 6 years or older

**Table 5:** Critical Quality Attributes (CQAs) definition and initial risk analysis for formulation. Where L: low, M: medium, and H: high.

CQA	Polymorph (Shape)	Particle Size (Distribution)	Moisture	Disintegrant	Lubricant
Blend					
Flow	M	H	L	H	H
Compression					
Hardness	H	M	L	H	M
Friability	H	M	L	H	M
Disintegration	L	L	L	H	L
Final risk	H	M	L	H	M

**Table 6:** SeDeM results for carbamazepine (API) and disintegrants L-HPC LH11, L-HPC NBD022, PARTECK^®^ ODT, and PROSOLV^®^ ODT.

				Carbamazepine		L-HPC L-H11		L-HPC NBD022		PARTECK^®^ ODT		PROSOLV^®^ ODT	
Incidence Factor	Parameter	Symbol	Units	Radius Value	Incidence	Radius Value	Incidence	Radius Value	Incidence	Radius Value	Incidence	Radius Value	Incidence
**Dimensions**	Bulk density	*Da*	g/mL	5.84	6.44	3.84	4.68	3.52	4.52	5.28	5.74	6.19	7.01
	Tapped density	*Dc*	g/mL	7.02		5.52		5.51		6.20		7.82	
**Compressibility**	Interparticle porosity	*Ie*	-	2.39	2.92	6.61	5.90	8.55	6.00	2.34	4.91	2.81	5.66
	Carr index	*IC*	%	3.36		6.09		7.22		2.97		4.17	
	Cohesion index	*Icd*	N	3.00		4.99		2.22		9.42		10.00	
**Flowability/Powder flow**	Hausner index	*IH*	-	8.99	7.28	7.81	3.00	7.18	3.42	9.13	7.30	8.69	7.26
	Angle of repose	*α*	°	4.69		1.19		3.10		4.60		5.09	
	Powder flow	*t*	s	8.17		0.00		0.00		8.17		8.00	
**Lubricity/Stability**	Loss on drying	*%HR*	%	9.67	9.81	3.10	4.93	6.02	4.95	8.12	8.90	6.18	7.98
	Hygroscopicity	*%H*	%	9.95		4.77		3.89		9.69		9.78	
**Lubricity/Dosage**	Particles < 50 µm	*%Pf*	µ	7.80	4.53	0.00	3.00	0.00	3.83	7.40	4.63	2.57	3.66
	Homogeneity index	*Iϴ*	-	1.25		6.00		7.65		1.85		4.75	
**Good compression index (IGC)**					5.72		4.12		4.35		5.96		6.03

**Table 7:** Average weight and hardness results for the proposed blends, corresponding to an improved compressibility factor (Formulation F3 was compressed at a different target weight).

Target Radius	Formulation	Mean Weight (mg)	Mean Hardness (N)	Tablet Diameter (mm)
**5**	F1	187.6	147.7	9
	F2	172.9	159.9	9
	F3	697.6	331.8	13
	F4	250.0	230.5	9
**4.5**	F5	160.9	121.9	8
	F6	112.0	113.4	8
	F7	172.7	48.0	8
	F8	131.3	78.3	8
**4.0**	F9	83.0	77.0	6
	F10	82.7	73.2	6
	F11	101.5	55.4	6
	F12	88.4	21.2	6
**3.5**	F13	67.0	62.5	5
	F14	62.8	24.8	5
	F15	69.9	27.6	5
	F16	68.1	37.4	5

**Table 8:** Characterization of the blend and tablets obtained for a compressibility radius of 3.5.

Formula	Hausner Ratio	Mean Hardness (N)	Friability (%)	Disintegration (s)	Tablet Diameter (mm)
**F13**	1.36	62.5	0.89	50	5
**F14**	1.32	24.8	0.38	45	5
**F15**	1.19	27.6	0.89	103	5
**F16**	1.23	37.4	1.00	420	5

**Table 9:** SeDeM characterization of Formulations F13 and F14.

				Formulation 13		Formulation 14	
Incidence factor	Parameter	Symbol	Units	Radius Value	Incidence	Radius Value	Incidence
**Dimensions**	Bulk density	*Da*	g/mL	4.84	5.72	4.73	5.61
	Tapped density	*Dc*	g/mL	6.59		6.49	
**Compressibility**	Interparticle porosity	*Ie*	-	4.58	6.63	4.78	6.73
	Carr index	*IC*	%	5.31		5.42	
	Cohesion index	*Icd*	N	10.00		10.00	
**Flowability/Powder flow**	Hausner index	*IH*	-	8.19	5.05	8.14	3.97
	Angle of repose	(*α*)	°	3.30		3.76	
	Powder flow	*t*	s	3.67		0.00	
**Lubricity/Stability**	Loss on drying	*%HR*	%	7.63	7.94	7.77	8.16
	Hygroscopicity	*%H*	%	8.24		8.55	
**Lubricity/Dosage**	Particles < 50 µm	*%Pf*	µ	2.69	2.27	0.62	1.28
	Homogeneity index	*Iϴ*	-	1.85		1.95	
**Good compression Index**		*ICG*	-	-	5.31	-	4.94

## Data Availability

Data are unavailable due to privacy restrictions.

## Supplementary Materials

The following supporting information can be downloaded at: https://www.mdpi.com/article/10.3390/pharmaceutics17050624/s1. Crystal X-ray assay was performed by the X-Ray Diffraction Service of CCiT of the Barcelona University. Figure S1: X-ray diffraction analysis performed on carbamazepine. Figure S2: Carbamazepine particle size distribution, batch 16CT000017.
