# Supplementary material for: Preformulation Study of Carbamazepine Orally Disintegrating Tablets for Pediatric Patients Using Direct Compression and the SeDeM Diagram Tool: A Quality by Design Approach
Source: Pharmaceutics. 2025 May 8;17(5):624. doi: 10.3390/pharmaceutics17050624 (PMC12115275; doi:10.3390/pharmaceutics17050624)

Preformulation Study of Carbamazepine Orally Disintegrating Tablets for Pediatric Patients Using Direct Compression and the SeDeM Diagram Tool: A Quality by Design Approach

Ricard Canadell-Heredia [1], Khadija Rouaz-El-Hajoui [1,2,*,†], Natalia Franco-Piedrahita [1], Pilar Pérez-Lozano [1,2], Marc Suñé-Pou [1,2], Josep María Suñé-Negre [1,2] and Encarna García-Montoya [1,2,*,†]

[1] Department of Pharmacy and Pharmaceutical Technology and Physical Chemistry, Faculty of Pharmacy and Food Sciences, University of Barcelona, Av. Joan XXIII, 27-31, 08028 Barcelona, Spain; rcanadellh@gmail.com (R.C.-H.); nataliai.francop@ub.edu (N.F.-P.); perezlo@ub.edu (P.P.-L.); marcsune@ub.edu (M.S.-P.); jmsune@ub.edu (J.M.S.-N.)

[2] Pharmacotherapy, Pharmacogenetics and Pharmaceutical Technology Research Group, Bellvitge Biomedical Research Institute (IDIBELL), Av. Gran via de l'Hospitalet, 199-203, 08090 Barcelona, Spain

* Correspondence: khadijarouaz@ub.edu (K.R.-E.-H.); encarnagarcia@ub.edu (E.G.-M.)

† These authors contributed equally to this work.

Abstract: Background/Objectives: Carbamazepine is widely used as a first-line treatment for pediatric patients with benign epilepsy. However, most commercial formulations have doses of 100 mg or higher, limiting their suitability for pediatric use. The aim of this study was to develop mini orally disintegrating tablets (ODTs) containing 50 mg of carbamazepine, utilizing direct compression technology, specifically tailored to meet the unique needs of pediatric patients. **Methods:** The development was carried out following a Quality by Design (QbD) approach, beginning with preformulation studies using the SeDeM expert system. Various co-processed excipients (PROSOLV® ODT and PARTECK® ODT) and non-co-processed excipients (L-HPC LH11 and L-HPC NBD-022) were evaluated. Additionally, modifications to the radius parameter of the SeDeM expert system were investigated to improve formulation design. **Results:** Optimized Formulations 13 and 14 achieved disintegration times below 1 min, hardness values between 25 and 60 N, and friability under 1%, fulfilling the predefined Critical Quality Attributes (CQAs). Tablets were successfully produced with a diameter of 5 mm and a weight below 100 mg. Moreover, reducing the SeDeM incidence radius from 5.0 to values between 4.0 and 3.5 proved viable, enabling the inclusion of excipients previously considered unsuitable and broadening formulation options without compromising quality. **Conclusions:** This study demonstrates the feasibility of producing small, fast-disintegrating, and mechanically robust 50 mg carbamazepine ODTs tailored for pediatric patients. It also validates the adjustment of SeDeM parameters as an effective strategy to expand excipient selection and enhance formulation flexibility in pediatric drug development.

Keywords: pediatric population; tablets; benign epilepsy; quality by design; SeDeM diagram

Figure S1. X-Ray diffraction analysis performed on carbamazepine.

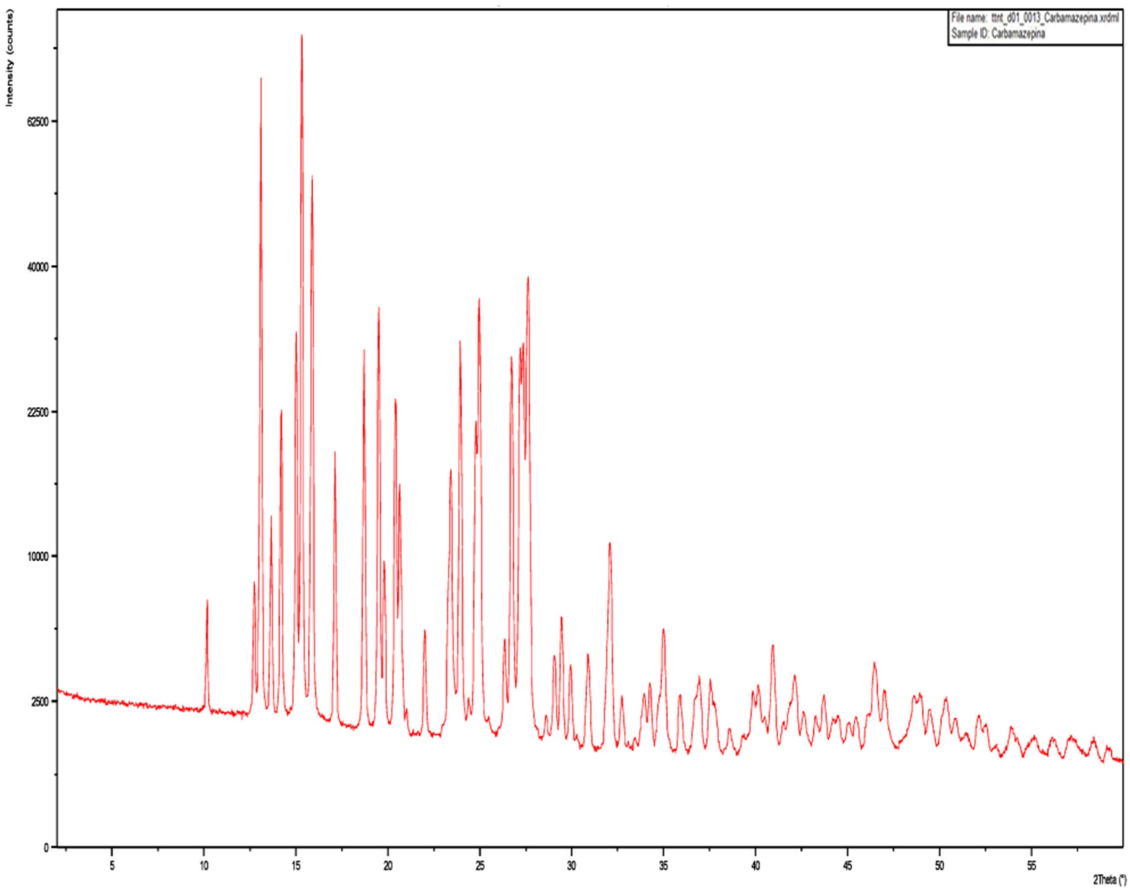

Figure S2. Carbamazepine particle size distribution, batch 16CT000017.

d(0.1): 6.958 um **d(0.5): 46.479 um** **d(0.9): 143.926 um**

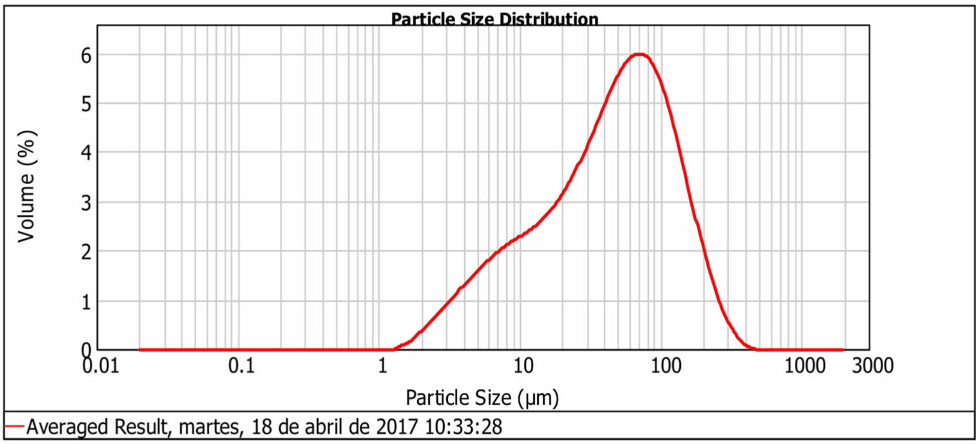

Supplement: Supplementary file 1 [file pharmaceutics-17-00624-s001.zip › pharmaceutics-3596232-supplementary.pdf]
